# When Hair Causes Harm: A Rare Case of Intestinal Bowel Obstruction by a Trichobezoar Mimicking an Acute Intestinal Intussusception

**DOI:** 10.7759/cureus.93329

**Published:** 2025-09-27

**Authors:** Kamal El Ghazy, Hind Cherrabi, Zineb Benmassaoud, Badr Rouijel, Mohamed Amine Oukhouya

**Affiliations:** 1 Pediatric Surgery Department, Souss Massa University Hospital Center, Faculty of Medicine and Pharmacy of Agadir, Ibn Zohr University, Agadir, MAR

**Keywords:** abdominal pain, enterotomy, intussusception, pediatric, trichobezoar

## Abstract

We report a rare case of a five-year-old girl with small bowel obstruction caused by an intestinal trichobezoar. The clinical picture was concerning due to the absence of symptom improvement and the emergence of bilious vomiting accompanied by progressive abdominal distention, raising suspicion for mechanical bowel obstruction. A rare and challenging clinical scenario arises when an intestinal trichobezoar mimics acute intestinal intussusception, leading to diagnostic confusion and potential treatment delays.

## Introduction

An intestinal bowel obstruction caused by a trichobezoar is a rare but significant clinical condition in children. Trichobezoars are masses formed from ingested hair, usually associated with underlying psychiatric disorders such as trichotillomania and trichophagia [1]. While they most commonly arise in the stomach, trichobezoars can extend into the small intestine or rarely lodge primarily there, leading to serious complications, including bowel obstruction [2]. One particularly challenging presentation occurs when a trichobezoar mimics acute intestinal intussusception, which shares similar clinical symptoms such as abdominal pain, vomiting, and palpable abdominal mass [3].

We report the case of a five-year-old girl hospitalized in the department of pediatric surgery at Universal Hospital Center of Sous Massa for an intestinal bowel obstruction by a trichobezoar mimicking an acute intestinal intussusception, highlighting the clinical features, diagnostic challenges, and essential role of CT imaging and surgical management.

## Case presentation

This is a case of a five-year-old girl with no psychiatric history, such as trichophagia and trichotillomania. The family was interviewed thoroughly about any relevant behaviors, and no history of hair-pulling or ingestion was reported. She was admitted to the emergency room due to diffuse abdominal pain and an obstructive syndrome that had been developing for five days. The general examination indicated that the patient was in good general condition, conscious, apyretic at 37.2 °C, and showed no visible signs of alopecia. The physical examination revealed a large, firm, mobile, painless, and non-pulsatile mass in the right iliac fossa.

The laboratory assessment found leukocytosis with neutrophil predominance at 16,000/ml; C-reactive protein (CRP) at 105 mg/l; and hyponatremia at 128 mmol/l was also noted (Table 1).

**Table 1:** Results of the laboratory tests

	Values of our case	Normal range [4]
Leukocytes (/μl)	16000	4800-10800
C-Reactive Protein (mg/l)	105	<3
Natremia (mmol/L)	128	135-145

**Figure 1:**
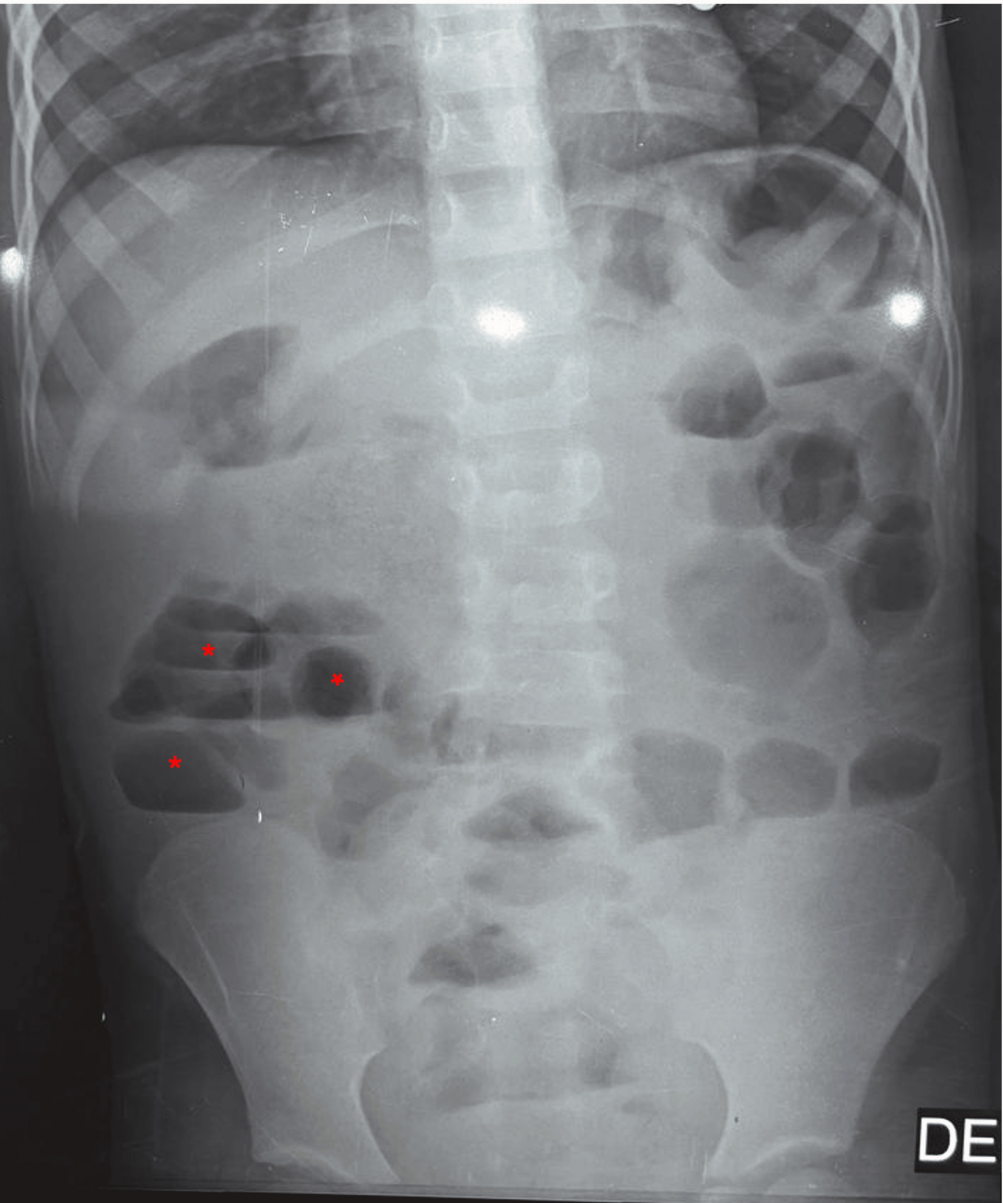
Upright abdominal X-ray showing small bowel air-fluid levels (red star)

An upright abdominal X-ray was obtained as part of the initial diagnostic workup to evaluate signs of bowel obstruction. The imaging demonstrated multiple air-fluid levels localized within the peripheral small bowel loops, a radiographic feature highly suggestive of mechanical intestinal obstruction (Figure 1).

**Figure 2:**
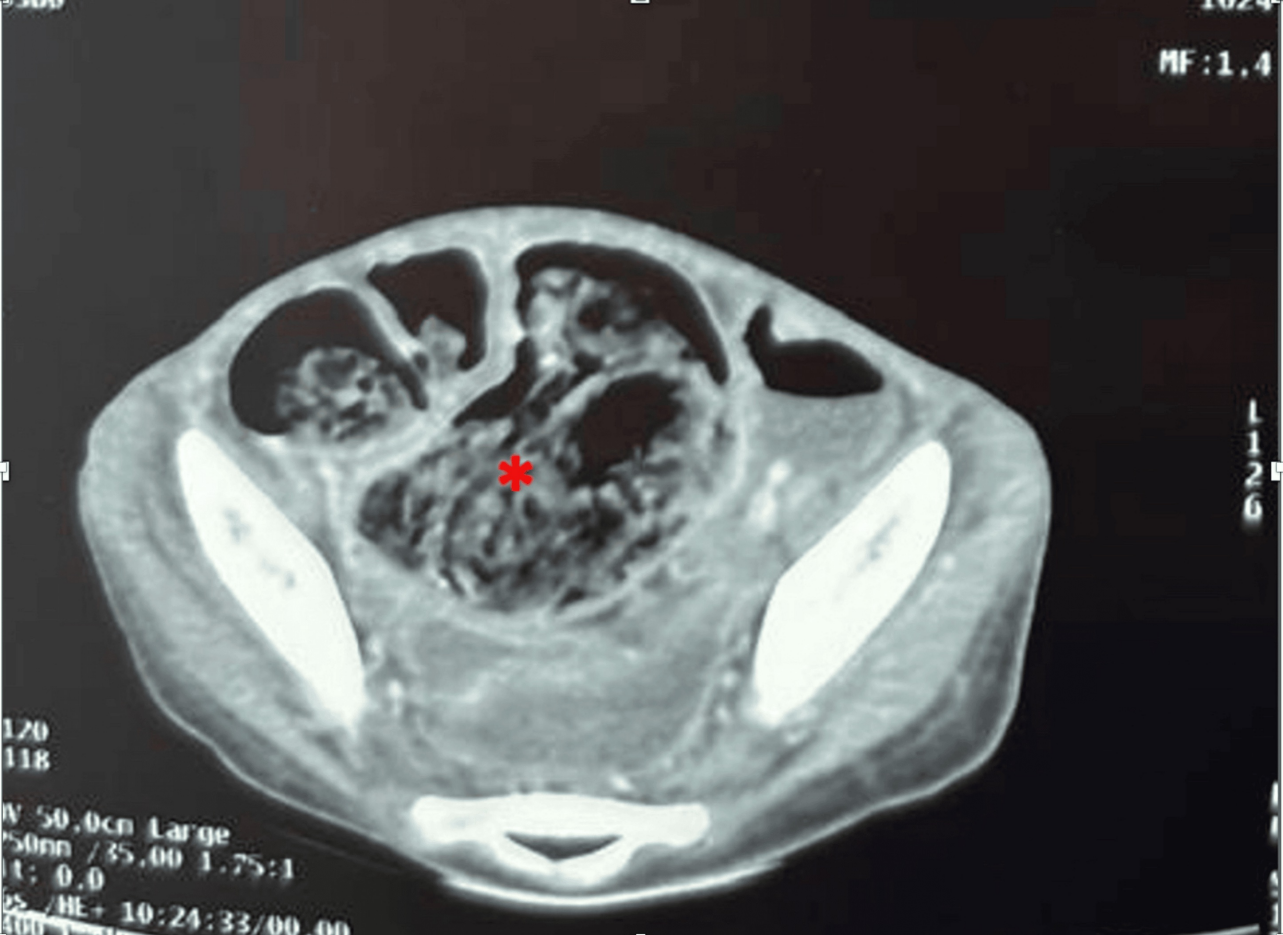
CT abdominal scan showing a pseudo-target-shaped, elongated, dense, and heterogeneous lesion in the distal ileum that does not enhance after contrast agent injection (red star)

A complementary computed tomography (CT) scan was performed to further investigate the cause of the patient’s symptoms and clarify the findings from the abdominal X-ray. The CT images revealed a distinctive, elongated, dense, and heterogeneous intraluminal mass in the distal ileal region. Importantly, this lesion exhibited no enhancement following intravenous contrast administration, suggesting a non-vascularized structure. On axial imaging, the mass demonstrated a characteristic pseudo-target or target-like appearance (Figure 2), which is often described in cases of acute intestinal intussusception due to the concentric layering of the intestinal walls involved in the telescoping process.

**Figure 3:**
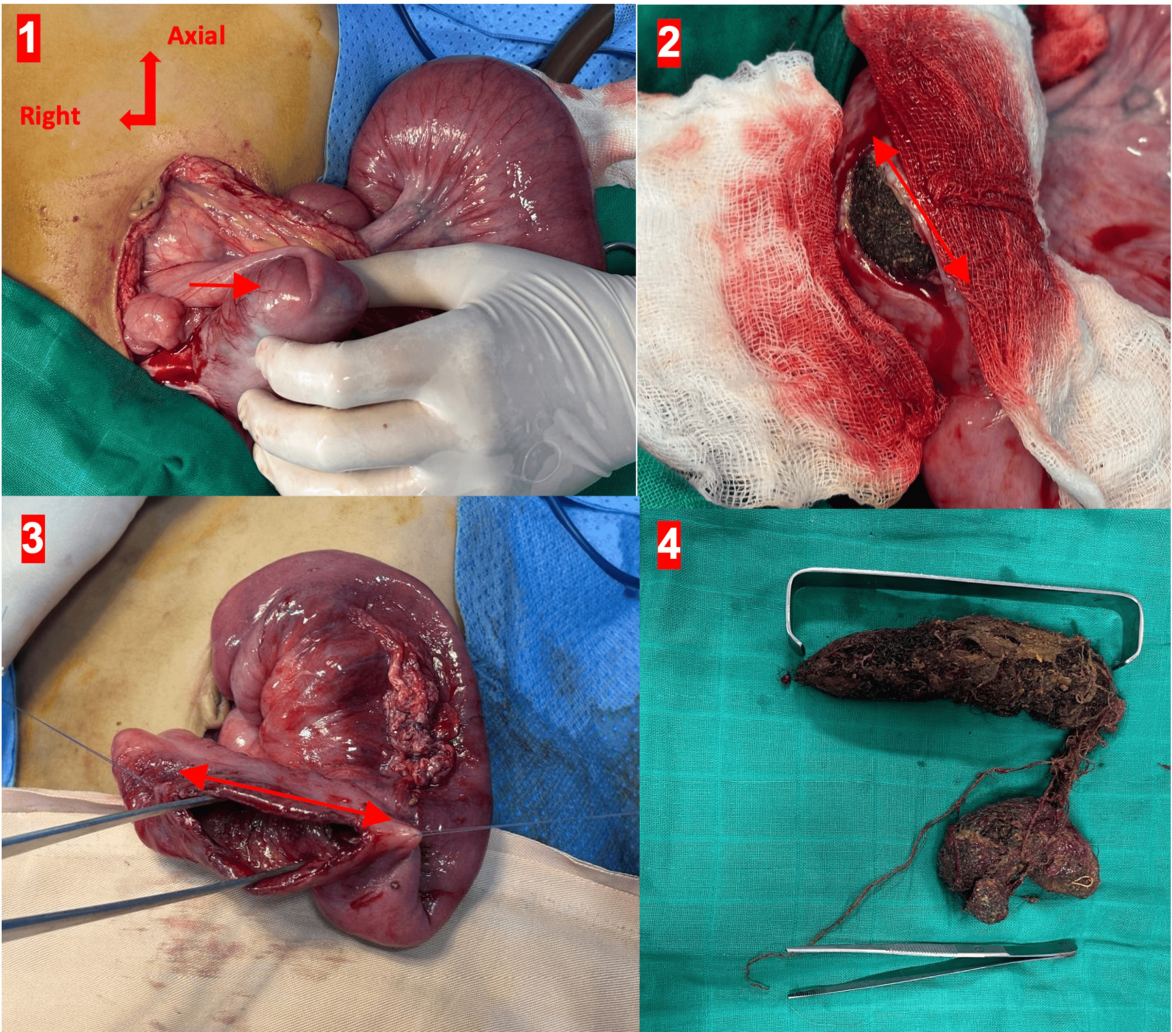
Mobile endoluminal mass moving away under the hand (1); Enterotomy allowing visualization of the trichobezoar (2,3); A large trichobezoar after extraction (4)

After ensuring the patient’s hydroelectrolytic balance was adequately stabilized, surgical intervention was undertaken via exploratory laparotomy to determine the cause of the bowel obstruction. Intraoperatively, no evidence of intestinal intussusception was observed, despite prior radiological suspicion. Careful palpation of the ileum revealed a freely movable intraluminal mass (Figure 3), raising suspicion for an obstructive foreign body. Consequently, an enterotomy was performed on the second ileal loop proximal to the ileocecal valve to access and remove the lesion. Surgical extraction yielded a large trichobezoar measuring approximately 15 cm in length, definitively confirming the diagnosis of bowel obstruction due to this uncommon bezoar. Gastric palpation did not identify a trichobezoar.

The surgical procedure led to complete resolution of the obstructive pathology, and the postoperative period was uneventful, with favorable clinical recovery. Given the underlying behavioral etiology implied by the trichobezoar, the patient was promptly referred for psychiatric evaluation and management to address potential trichotillomania or related conditions and to prevent recurrence.

## Discussion

Trichobezoars are predominantly seen in pediatric and adolescent populations, often linked to psychiatric disorders, including trichotillomania and trichophagia. They primarily localize within the stomach but may occasionally extend into the jejunum, ileum, and colon [5].

The clinical presentation and physical examination findings vary according to the size and anatomical location of the trichobezoar, as well as any associated complications [6]. In the initial stages, patients are often asymptomatic with no evident pathological signs. As the bezoar increases in size, symptoms such as abdominal pain, vomiting, abdominal distension, early satiety, weight loss, and constipation may develop. As obstruction progresses, postprandial vomiting and colicky abdominal pain may occur [7].

Computed tomography (CT) is the most valuable diagnostic modality for patients with bezoars, as it accurately identifies both the location and nature of bowel obstruction [8]. On CT imaging, an enteric bezoar typically appears as a dilated small bowel loop containing a well-defined, round or ovoid, heterogeneous intraluminal mass at the transition point where the obstruction occurs [9]. This characteristic appearance helps differentiate bezoars from other causes of bowel obstruction.

In some cases, a trichobezoar can mimic acute intestinal intussusception on imaging studies. This phenomenon, sometimes referred to as “trichobezoar mimicking intussusception,” occurs because the hair mass can cause the telescoping of one part of the intestine into another, leading to similar clinical and radiological features as true intussusception [10]. Recognizing this possibility is crucial because management differs; while intussusception often requires urgent surgical reduction, trichobezoars may require targeted removal, typically through surgical or endoscopic extraction. Thus, CT not only confirms the presence of a trichobezoar but also assists in avoiding misdiagnosis with conditions like intussusception, guiding appropriate therapeutic intervention. Baheti et al. report cases where trichobezoars cause small bowel intussusception visible on CT scans, illustrating this rare complication and the diagnostic challenge it poses. This condition, sometimes referred to as “trichobezoar-induced intussusception,” has been documented in children and requires careful imaging assessment to distinguish it from true intussusception [11].

Laparotomy plays a crucial role in the management of children presenting with trichobezoar mimicking acute intussusception, especially when noninvasive diagnostic methods are inconclusive or when there is a risk of bowel obstruction or ischemia [12]. In such cases, the clinical and radiological presentation may closely resemble true intussusception, leading to diagnostic uncertainty. When imaging studies, such as CT, suggest a bezoar causing bowel obstruction or intussusception-like symptoms, laparotomy allows direct visualization and definitive treatment [13]. This approach enables the safe removal of the bezoar mass, often by enterotomy or segmental resection if bowel viability is compromised. Additionally, laparotomy provides the opportunity to inspect the entire gastrointestinal tract for additional bezoars, which might not be detected on imaging, thus preventing recurrent obstruction.

Overall, laparotomy serves both a diagnostic and therapeutic role in cases of trichobezoar-induced acute intestinal obstruction mimicking intussusception, offering a definitive solution while minimizing the risk of complications [14]. Early surgical intervention through laparotomy is often associated with favorable outcomes, reducing morbidity and preventing recurrent episodes in these complex pediatric presentations [15].

## Conclusions

The combination of detailed CT imaging and timely laparotomy ensures the accurate diagnosis and effective management of trichobezoar-induced intestinal obstruction mimicking acute intussusception. This approach minimizes complications and promotes favorable outcomes, while postoperative psychiatric evaluation and therapy are crucial to prevent recurrence associated with behavioral disorders underlying trichobezoar formation.

## References

[1] Kaba M, Karadağ ÇA, Sever N, Ser İ, Demir M, Yıldız A, Usta AM (2023). A rare cause of intestinal obstruction in children trichobezoar: how to diagnose?. Ulus Travma Acil Cerrahi Derg.

[2] Ezoddin N, Sobhanian P, Mousavi SA, Reisi N ( 2025). Management of gastric trichobezoar in children: a case report and literature review. J Pediatr Rev.

[3] Won MM, Sacks MA, Leigh R, Mendez YS, Goodman LF, Tagge E, Radulescu A (2022). An unusual case of primary ileal trichobezoar causing intussusception. Am J Case Rep.

[4] American Board of Internal Medicine (2025). American Board of Internal Medicine. ABIM laboratory test reference ranges-January 2025. american board of internal medicine.

[5] Wang L, Chen Y, Chen S, Gao Z, Qian Y, Chen Q (2024). Gastrointestinal trichobezoars in the pediatric population: a retrospective study. BMC Pediatr.

[6] Mirza MB, Talat N, Saleem M (2020). Gastrointestinal trichobezoar: an experience with 17 cases. J Pediatr Surg.

[7] Goyal V, Goyal PK, Gupta M (2014). A rare case of small bowel obstruction due to primary trichobezoar. J Clin Diagn Res.

[8] Lee JM, Jung SE, Lee KY (2002). Small-bowel obstruction caused by phytobezoar: MR imaging findings. AJR Am J Roentgenol.

[9] García-Ramírez BE, Nuño-Guzmán CM, Zaragoza-Carrillo RE, Salado-Rentería H, Gómez-Abarca A, Corona JL (2018). Small-bowel obstruction secondary to ileal trichobezoar in a patient with Rapunzel syndrome. Case Rep Gastroenterol.

[10] Singh S, Wakhlu A, Pandey A, Gupta A, Ahmed I, Chandra N (2011). Complicated Rapunzel syndrome mimicking intussusception. BMJ Case Rep.

[11] Baheti AD, Otjen JP, Phillips GS (2017). A hairy situation: trichobezoar presenting with intussusception, and intestinal and biliary perforation in a child. Radiol Case Rep.

[12] Gorter RR, Kneepkens CM, Mattens EC, Aronson DC, Heij HA (2010). Management of trichobezoar: case report and literature review. Pediatr Surg Int.

[13] Chintamani Chintamani, Durkhure R, Singh JP, Singhal V (2003). Cotton Bezoar--a rare cause of intestinal obstruction: case report. BMC Surg.

[14] Kumar VS, Shenoy AM, DCunha AR, Kumar S, Shenoy RD (2024). Trichobezoars in children - a psychological perspective. Asian J Psychiatr.

[15] Almouallem MM, Hanna M, Martini N, Alfarouh A, Mousseli R, Yaldany M, Mahmod J (2025). Trichobezoar as an unusual cause for iron deficiency anemia: a rare case report. Clin Case Rep.

